# Wetting Behavior and Maximum Retention of Aqueous Surfactant Solutions on Tea Leaves

**DOI:** 10.3390/molecules24112094

**Published:** 2019-06-01

**Authors:** Feng Zhu, Chong Cao, Lidong Cao, Fengmin Li, Fengpei Du, Qiliang Huang

**Affiliations:** 1 Institute of Plant Protection, Chinese Academy of Agricultural Sciences, Beijing 100193, China; gzzbszf@163.com (F.Z.); ccao@ippcaas.cn (C.C.); caolidong@caas.cn (L.C.); fmli@ippcaas.cn (F.L.); 2 Institute of Plant Protection, Guizhou Academy of Agricultural Sciences, Guiyang 550006, China; 3 Department of Applied Chemistry, College of Science, China Agricultural University, Beijing 100193, China

**Keywords:** wetting behavior, maximum retention, tea leaves, surfactants

## Abstract

In this research, the maximum retention and wetting behavior of surfactant solutions (N-200, N-300, Tween-80, Morwet EFW, DTAB, SDS) on the surfaces of tea leaves was investigated based on surface free energy, surface tension, the contact angle, adhesion work, and adhesion force. The results showed that the contact angles of all surfactant solutions were kept constant with low adsorption at the tea leaf–liquid interfaces below 0.005%. With an increase in concentration, the contact angle of Tween-80 decreased sharply because the adsorption of molecules at the solid–liquid interfaces (*Γ_SL’_*) was several times greater than that at the liquid–air interfaces (*Γ_LV_*). Adhesion work decreased sharply and then reached a minimum at the critical micelle concentration (CMC), but then increased until reaching a constant. Moreover, a high adhesion force did not indicate better wettability, as it does with rose petals and peanut leaves. For tea leaf surfaces, an increase in the contact angle brought about an increase in the adhesion force. In addition, the maximum retention for Morwet EFW is at different concentrations compared to N-200, N-300, Tween-80, DTAB, and SDS, where the maximum retention of Morwet EFW on tea leaves was 6.05 mg/cm^2^ at 0.005%.According to the mechanisms of wetting behavior on plant surfaces, a recipe for pesticide formulation can be adjusted with better wettability to reduce loss, improve utilization efficiency, and alleviate adverse effects on the environment.

## 1. Introduction

Plants always suffer from attacks by biological factors, such as pests, plant diseases, and weeds. In the wild, pesticides are considered to be one of the most effective ways to protect plants from harmful organisms. However, it is not as easy to control pesticides when they are applied in a natural environment compared to in a laboratory. In order to improve the efficiency of pesticides, excessive amounts of pesticides are applied, so the food and the environment may become polluted by pesticide residue. Therefore, it is essential to take the method of pesticide utilization into account.

The efficiency of pesticides on plants is affected by various factors. Apart from the active ingredient, the behavior of pesticide solutions on plant surfaces may influence control efficiency due to the bouncing and splashing of pesticide droplets [1]. This means that the efficiency of pesticides is affected by its retention on plant leaf surfaces, and it is especially closely related to the poor wettability of pesticide droplets on plant leaf surfaces [2]. Wetting behavior has attracted significant attention due to its wide applications in coating, gluing, and agriculture [3,4]. Therefore, understanding the wetting behavior of pesticide solutions is one of the most important factors in improving the efficacy of pesticides. Generally, the wetting characteristics of a solid surface can be classified into three types. The first is a hydrophilic solid when the contact angle (*θ*) of a droplet on the solid surface is lower than 90°. In this case, plant leaf surfaces can be easily wetted by pesticide droplets, so little pesticide will bounce from the leaf surface. The second is a hydrophobic solid surface, when *θ* is above 90°. However, when *θ* is over 160°, the surface is considered to be superhydrophobic [5]. Most plant leaf surfaces are different from each other: Furthermore, pesticide formulations are not based on the characteristics of plant leaf surfaces when they are prepared [6]. Thus, some plant leaf surfaces are hard to wet due to the poor wettability of pesticide solutions. Consequently, the loss of pesticides in agricultural production causes unwanted problems, including negative environmental and ecological effects, as well as effects on public health. Recently, the side effects of pesticides have attracted considerable interest: More and more researchers are attempting to reduce the amount of pesticides used and improve their efficiency. While plant leaf surfaces cannot be changed artificially, effective and simple approaches to improving the wettability of pesticide droplets mainly involve amending the liquid properties of pesticides by adding surfactants into pesticide formulations [7,8]. Apart from the fact that surface free energy (SFE) influences solid surfaces(i.e., solid surfaces that are hard to wet always have a low surface free energy) [4], the aforementioned approach is considered to be a simple method because surfactants can help to reduce the surface tension of pesticide solutions.

To date, the wetting behavior of surfactant solutions on abiotic solid surfaces has been extensively researched [9,10,11,12,13]. In comparison to abiotic solid surfaces, the wetting behavior of plant surfaces is more complicated, as seen in wheat [14], ginkgo leaves [15], and rice [16]. It is very common to add a surfactant into a pesticide to obtain controllable efficiency by improving the solution wettability on a given plant. For example, for hydrophobic plants, such as rice and some weed leaves, pesticide efficacy can be improved significantly after the solution is adjusted by a surfactant. Tea plants are commonly found in China. Compared to other agricultural plants, tea leaves are used to make one of the most important drinks around the world, and especially in China. However, tea plants differ from other agricultural plants, because tea leaves are consumed by the public directly after a simple processing method carried out in China. Thus, apart from the efficiency of pesticides, it is crucial to regulate pesticide residue in tea. In addition, the wetting behavior of the surfactant can be greatly influenced by the plant leaf surface. Therefore, understanding the wetting behavior of the surfactant on tea leaves is one method to ensure good tea production and food safety.

Tea is a typical plant, the leaf surface is the part of the plant most vulnerable to pests and diseases. However, as tea is consumed daily in China, the pesticide residues in tea should be reduced as much as possible. Therefore, leaf surface properties should be clarified, and the properties of surfactant solutions should be precisely regulated so that pesticide droplets can be deposited and retained on tea leaf surfaces as much as possible. Higher doses of solutions can meet the needs of pest control dosage and reduce the risk of residues, because the higher the pesticide solution dosage that can stick to tea leaf surfaces, the lower the concentration of pesticides required and the lower the risk of residue in tea. In addition, by increasing the retention of pesticide solutions on leaf surfaces, the loss of chemicals can be reduced, thus reducing the harm to beneficial organisms and the environment as well as reducing the risk of residual chemicals being transmitted upward to the leaf surface through root absorption. Therefore, in this study, we determined the hydrophobic and hydrophobic properties of the tea interface by testing their respective indicators, and we clarified the surface properties of six surfactants. Based on the properties of tea leaf surfaces and surfactant solutions, combined with effective retention on tea leaf surfaces, accurate control over surfactants for pest and disease prevention was achieved.

Generally, the use of various amounts of surfactants leads to different wetting abilities. Retention was measured through the immersion method. Note that for tea leaf surfaces, the application of greater amounts of pesticide does not lead to high efficiency. It is more complicated in field conditions because the solution will roll down the plants, leading to spray run-off. Thus, obtaining a measure of the maximum retention is crucial to determining the optimum recipe of a pesticide. Moreover, the maximum retention is related to the theoretical retention factor, which is calculated from the values of the contact angles, adhesion force, adhesion work, and so on, as well as from the surface tension of the spray liquid. In the present study, the wetting behavior of surfactant solutions was evaluated with six different commercial surfactant solutions. To characterize the wetting behavior on tea leaf surfaces, the surface free energy, surface tension, contact angle, and adhesion force were measured. The results showed that the wetting processes of the surfactants on tea leaf surfaces were different from each other, which is very useful in evaluating which surfactant should be added into pesticide formulations. This research may aid in understanding wetting behavior on tea leaf surfaces. Based on this work, potential methods to develop new pesticide formulations are provided, so that the efficiency of pesticides applied to tea plants can be improved and the side effects of the pesticides can be minimized so as to ensure food safety when tea is consumed by the public.

## 2. Experiments and Materials

### 2.1. Materials

The polymeric surfactantsN-200 (acrylate homologues) and N-300 (aliphatic alcohol polyoxyethyleneethers) were produced by the Dauni Research Center of Advanced Science and Technology Co., Ltd. (Shantou, China). Morwet EFW(blend of alkyl naphthalene sulfonate and anionic wetting agent) were produced by Azkonobel (Compton, CA, USA). Tween-80 (Polyethylene glycol sorbitan monooleate) C_24_H_44_O_6_[C_2_H_4_O]_n_ was purchased from Solarbio Life Science (Beijing, China). Sodium dodecyl sulfate (SDS) [C_12_H_25_SO_4_Na] and dodecyl trimethyl ammonium bromide (DTAB) [C_15_H_34_BrN] were purchased from Sigma-Aldrich Co., Ltd. (Shanghai, China), as shown in Table 1. All of the surfactants were used for the preparation of aqueous solutions without further purification. Aqueous solutions of the individual surfactants were prepared using Milli-Q water, and the concentrations were in the range of 0.0001% to 1%.

Tea leaves were collected from the tea base of the Institute of Tea of the Guizhou Academy of Agricultural Sciences (Guiyang, China). The length and width of each leaf was strictly controlled. All leaves were cut into strips, avoiding breakage of the middle vein, and were then attached to a glass slide. The SFE of each sample was calculated by the Owens, Wendt, Rabel, and Kaelble (OWRK) method using four test liquids (water (Milli-Q), formamide, ethylene glycol, and dimethyl formamide (Sinopharm Chemical Reagent Co., Ltd., Shanghai, China).

### 2.2. Microscopy

Tea leaves were washed with 0.1 M of phosphate buffer (pH = 7.2) for 20 min, and dehydration of the leaves was achieved with different concentrations of ethanol (from 30% to 100%) seven times. Then the samples were dried using the critical drying method. Several pieces of tea leaves were put on the specimen stage with double-sided adhesive tape. After that, the samples were placed into an ion sputtering apparatus for gold spraying. To determine the microstructures of tea leaf surfaces, the prepared specimens were observed by scanning electron microscopy (SEM) (JEM-1400, JEOL Ltd., Tokyo, Japan).

### 2.3. Evaluation of the Surface Free Energy

In the OWRK method [16], it is assumed that SFE is formed by dispersive and polar components on the basis of the Berthelot hypothesis. Using the contact angles of four test liquids on tea leaf surfaces, the SFE of tea leaf surfaces can be calculated according to Equation (1):

(1) $$\frac{\left(1+\cos\;\theta \right)\cdot \gamma_{LV}}{2\sqrt{\gamma_{LV}^{d}}}=\sqrt{\gamma_{SV}^{p}}\times \sqrt{\frac{\gamma_{LV}^{p}}{\gamma_{LV}^{d}}}+\sqrt{\gamma_{SV}^{d}},$$

where $\gamma_{LV}$, $\gamma_{LV}^{d}$*,* and $\gamma_{LV}^{p}$ represent the surface tension, the dispersive parts, and the polar parts of the liquid, respectively; and $\gamma_{SV}^{d}$ and $\gamma_{SV}^{p}$ represent the dispersive and polar parts of the solid, respectively. According to Equation (1), by plotting $\frac{\left(1+\cos\theta \right)\cdot \gamma_{LV}}{2\sqrt{\gamma_{LV}^{d}}}$ versus $\sqrt{\frac{\gamma_{LV}^{p}}{\gamma_{LV}^{d}}}$, $\gamma_{SV}^{d}$ and $\gamma_{SV}^{p}$ can be calculated from the intercept and the slope, respectively. The value of $\gamma_{SV}$ is the sum of $\gamma_{SV}^{d}$ and $\gamma_{SV}^{p}$.

### 2.4. Surface Tension Measurements

The surface tension of different surfactant solutions was measured by the Wilhelmy plate method using a tensiometer DCAT 21 (DataPhysics Instruments GmbH, Filderstadt, Germany) at 298 ± 0.1 K. The platinum plate was burned under an alcohol flame after being washed by deionized water and alcohol to remove impurities before each measurement. To guarantee the cleanliness of the plate, the surface tension of water was used as a control to calibrate the tensiometer. The measurements were conducted until the surface tension values remained unchanged. More than 10 successive measurements were carried out, and the standard deviation did not exceed ±0.20 mN m^−1^.

### 2.5. Contact Angle Measurements

Measurements of the contact angles for pure solvents and surfactant solutions on leaf surfaces were carried out via the sessile drop method using a contact angle measuring system OCA 20 (DataPhysics Instruments GmbH, Filderstadt, Germany) at 298 ± 0.1 K, with a relative humidity around 65%. In brief, 3 μL of droplets were taken up with asyringe and immediately deposited onto the surface (within 60 s). The measurements were repeated 10 times. The results were analyzed by SCATsoftware 5.0.24 (DataPhysics Instruments GmbH, Filderstadt, Germany).

### 2.6. Adhesion Force Measurements

Measurements of force based on the pressing and pulling off of liquid droplets on tea leaf surfaces was achieved using a DCAT 21 (Data Physics) at 298 ± 0.1 K, with a relative humidity around 65%. In brief, 10 μL of droplets were syringed on the ring. The measurements were repeated 10 times.

### 2.7. Maximum Retention Measurements

The leaf area (s, cm^2^) of fresh tea leaves was measured by a leaf area measuring instrument (Yaxin-1242, Beijing Yaxin Science Instrument Technology Co., Ltd., Beijing, China), and the weight (m_1_, g) of each leaf was measured by an electronic balance (DT-502, Changshu Yiou Instrument Co., Ltd., Changshu, China).Then, each leaf was immersed into different concentrations of surfactant solutions for 5 s using tweezers. After that, the weight of leaves (m_2_, g) was measured until the drops did not fall down from the leaves after dipping them into the surfactant solutions. This procedure was repeated 10 times, and the results were averaged. According to the weight change of tea leaves before and after soaking, the maximum retention (*R*_m,_ mg/cm^2^) on tea leaves was calculated according to the following equation:

*R*_m_ (mg/cm^2^) and *R*_m_ = (m_2_ − m_1_) × 1000/s × 100. (2)

## 3. Results and Discussions

### 3.1. Tea Leaf Surfaces

**Figure 1:**
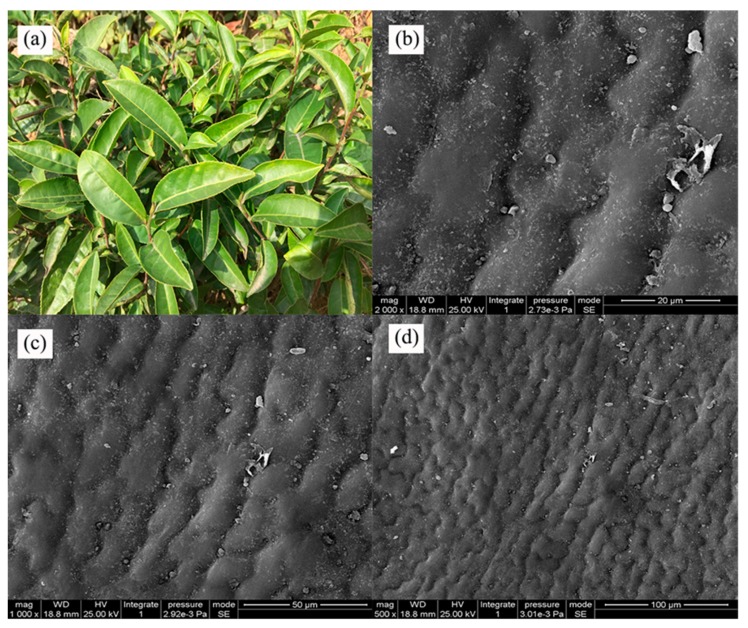
Tea leaves (**a**); scanning electron microscopy images of tea leaf surfaces (**b**–**d**).

As shown in Figure 1, the appearance of tea leaves (Figure 1a) was glossy, similar to *Magnolia grandiflora*, the surface of which has a flat wax film [17]. As shown in the SEM images (Figure 1b,c), the undulating surfaces of tea leaves were apparent. However, these observations differed from most hydrophobic plants, which are typically covered by fine nanostructures, bumps, or deep pitches, all of which contribute significantly to the quality of superhydrophobicity.

### 3.2. Characteristics of Tea Leaf Surfaces and Aqueous Surfactant Solutions

**Figure 2:**
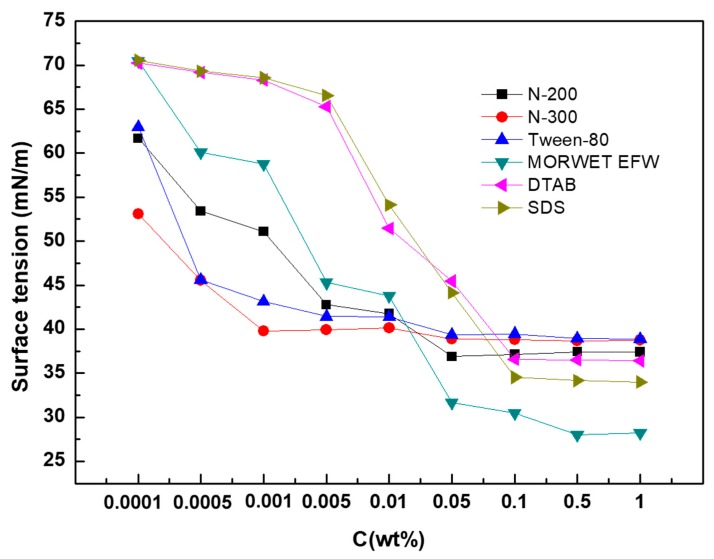
Surface tensions of aqueous surfactant solutions.

The SFE of the tea leaf surfaces was calculated by the OWRK method using the contact angle of four test liquids on leaf surfaces, as shown in Table 2. As shown in Table 3, the SFE of tea leaves was about 30.4 mJ m^−2^, which is similar to that of the moderately hydrophobic surface of polymethyl methacrylate (PMMA) (33.13 mJ m^−2^). As shown in Figure 2, the surface tensions of the N-200, N-300, Tween-80, Morwet EFW, DTAB, and SDS solutions decreased gradually with an increase in the concentration (The dynamic surface tensions for SDS solutions is shown in Supplementary Materials Figure S1). Five of them exhibited the same critical micelle concentration (CMC), 0.1 wt%, while that of Morwet EFW was different. Compared to the other tested surfactants, the surface tension of Morwet EFW was about 28.0 mN/m.

### 3.3. Wettability of Tea Leaf Surfaces

**Figure 3:**
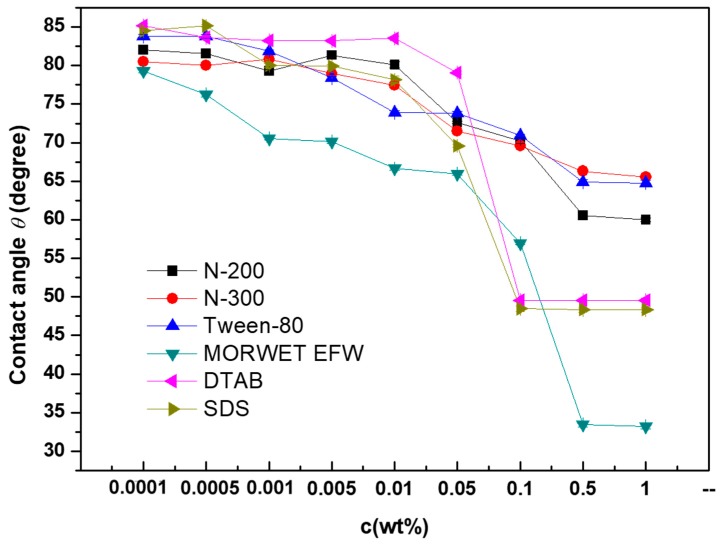
The relationship between the values of the contact angle (*θ*) on tea leaf surfaces and the surfactant concentration (*C*, wt%).

**Figure 4:**
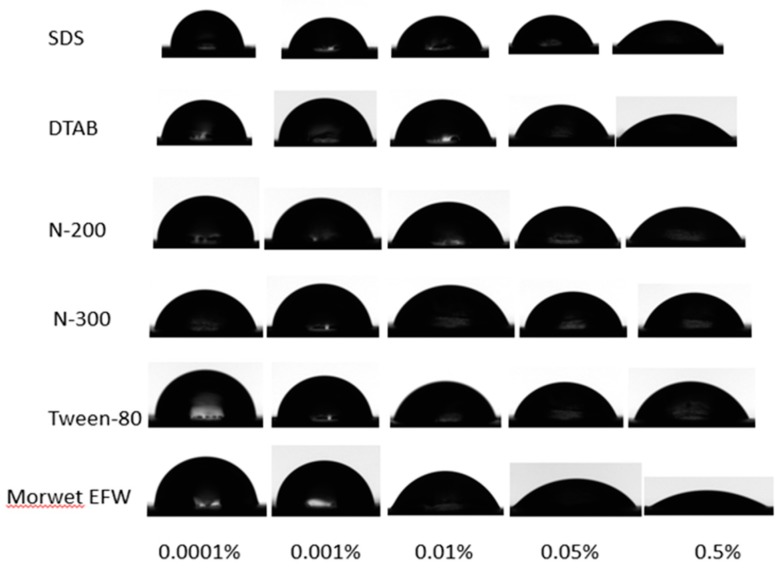
Optical images of contact angles on tea leaf surfaces according to the wetting stage.

**Figure 5:**
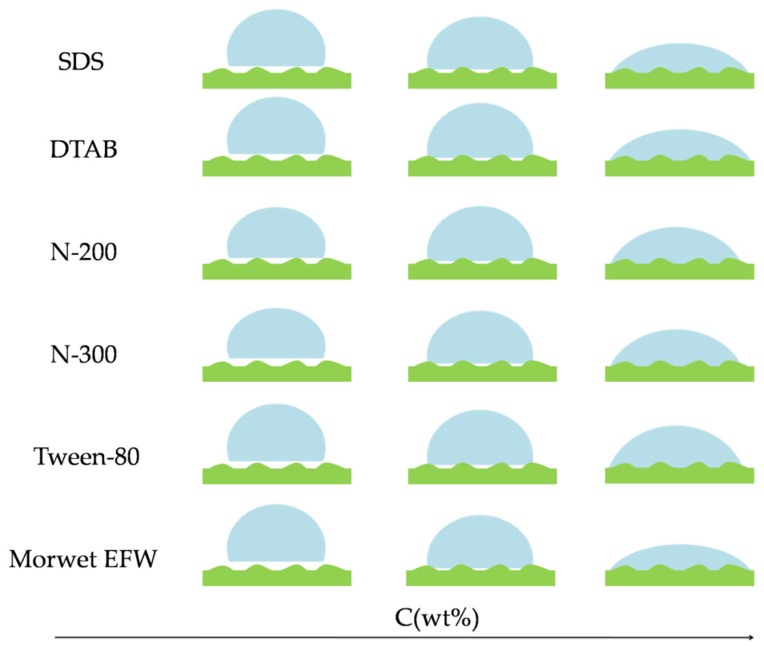
Schematic models of surfactant molecules of SDS, DTAB, N-200, N-300, Tween-80, and Morwet EFW adsorbed at the tea leaf surfaces.

The values of the contact angles for aqueous surfactant solutions versus the mass concentrations are shown in Figure 3. With the concentrations between 0.0001% and 0.01%, the contact angles of Tween-80, N-200, and N-300 remained constant even as the surface tension decreased gradually. However, when the concentration reached up to 0.01%, the contact angle decreased gradually until the concentration reached 0.05%. Compared to Tween-80, N-200, and N-300, with the increasing concentration of DTAB, SDS, and Morwet EFW, the contact angle gradually decreased until the concentration reached 0.05%. Then the contact angle decreased dramatically in the range of 0.05% to 0.1%, and finally the contact angle reached a plateau. However, when the mass concentration was above 0.5%, the contact angle of Morwet EFW on tea leaf surfaces decreased below 35° and remained at a balance phase, demonstrating that the commercial surfactant has a relatively good surface activity on tea leaf surfaces. For tea leaves, as shown in Figure 4, due to their undulating surfaces, the contact angle decreased with an increase in the concentration. As shown in Figure 5, the tea leaf surfaces could not be wetted by surfactant solutions completely in a Cassie–Baxter state. Upon increasing the concentration, the value of the contact angle decreased sharply in the transition from a Cassie–Baxter state to a Wenzel state. However, this state depends on the size and geometry of the leaf surface as well as the size of the droplet [21]. In fact, many factors affect wettability, such as wax and plant leaf morphologies [22], but the impact of wax is relatively small compared to the impact of surface structure on wettability [17]. Moreover, the contact angle can be dramatically affected by the presence of roughness [23,24]: The greater the roughness, the lower the wettability [25].

### 3.4. Adsorption at Solid–Liquid and Liquid–Air Interfaces

In the past, many studies on the wettability of leaf surfaces have employed the contact angles of surfactant solutions or water, as well as the results of cos*θ* versus *γ_LV_* plots to cos*θ* = 1. This allowed us to estimate the critical surface tension of wetting (*γ_C_*). Although there is no linear dependence between cos*θ* and *γ_LV_*, the linear relationship between the surface tension(*γ_LV_*) and adhesion tension (*γ_LV_*cos*θ*) can be found according to the method of Bargeman and van Voorst Vader:

*γ_LV_*cos*θ* = a*γ_LV_* + b (3)

where a and b are constants. Unlike ideal surfaces, there is no linear dependence between the surface tension of surfactant solution (*γ_LV_*) and the adhesion tension (*γ_LV_*cos*θ*) over the whole range for tea leaf surfaces.

The interfacial tension of the solid–liquid interface can be changed by the absorption of surfactant molecules at the surface, but there is no direct method to measure interfacial tension. In the present study, based on the surface tension *γ_LV_*, the contact angle of surfactant solutions on tea leaf surfaces, and the surface free energy, the interfacial tension could be obtained using Young’s equation (Equation (4)):

(4) $$\gamma_{LV}\cos\theta =\gamma_{SV}-\gamma_{SL}$$

where $\gamma_{SV}$, $\gamma_{SL}$, and $\gamma_{LV}$ represent the interfacial tension at the solid–air, solid–liquid, and liquid–air interfaces, respectively.

Combining Young’s equation and Gibbs’s formula, Lucassen-Reynders et al. [26] suggested that the relative adsorption at the interface can be determined by the relationship between *γ_LV_*cos*θ* and *γ_LV_*:

(5) $$\frac{d\left(\gamma_{LV}\cos\theta \right)}{{d\gamma }_{LV}}=\frac{\Gamma_{SV}-\Gamma_{SL}}{\Gamma_{LV}}$$

where $\Gamma_{SV}$, $\Gamma_{SL}$*,* and $\Gamma_{LV}$ represent the surface excess concentration of surfactants at the solid–air, solid–liquid, and liquid–air interfaces, respectively. Normally, when *Γ_SV_≈* 0, it is possible to establish the ratio of $\Gamma_{SL}$ to $\Gamma_{LV}$, which can be obtained by plotting *γ_LV_*cos*θ* versus $\gamma_{LV}$.

**Figure 6:**
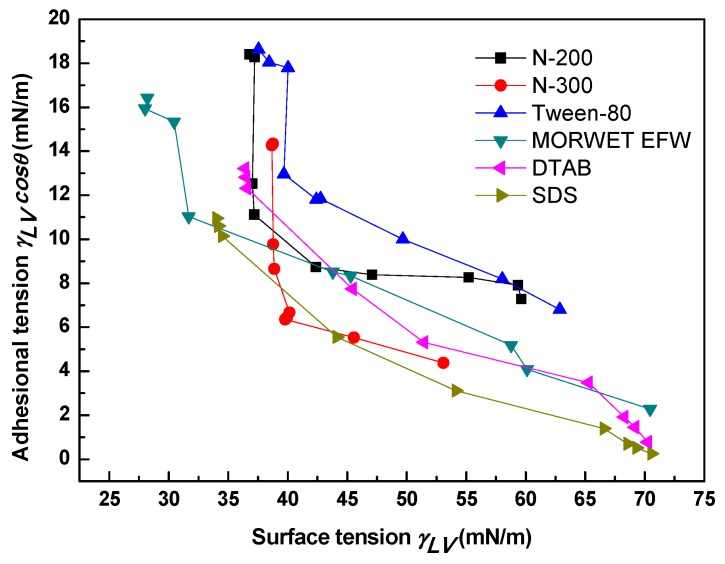
Relationship between surface tension (*γ_LV_*) and adhesion tension (*γ_LV_*cos*θ*) on tea leaf surfaces.

The relationship between *γ_LV_*cos*θ* and *γ_LV_*is shown in Figure 6, and it can be divided into two stages. In the first stage, N-200, N-300, DTAB, SDS, and Morwet EFW concentrations corresponded to values lower than 37.19, 39.95, 7.74, 3.11, and 31.67 mN/m, and the *γ_LV_*cos*θ* values decreased sharply, which could not be described by a linear function. Compared to the above five surfactants, the variation trend of Tween-80 could be fitted by a linear function. All of the slope and coefficient values obtained from the relationship between *γ_LV_*cos*θ* and *γ_LV_*are listed in Table 4. It is obvious that the absolute values of the slope were less than 1, which suggests that the volume of the absorbed surfactant molecules at the vapor–liquid interface was higher than that at the solid–liquid interface.

**Figure 7:**
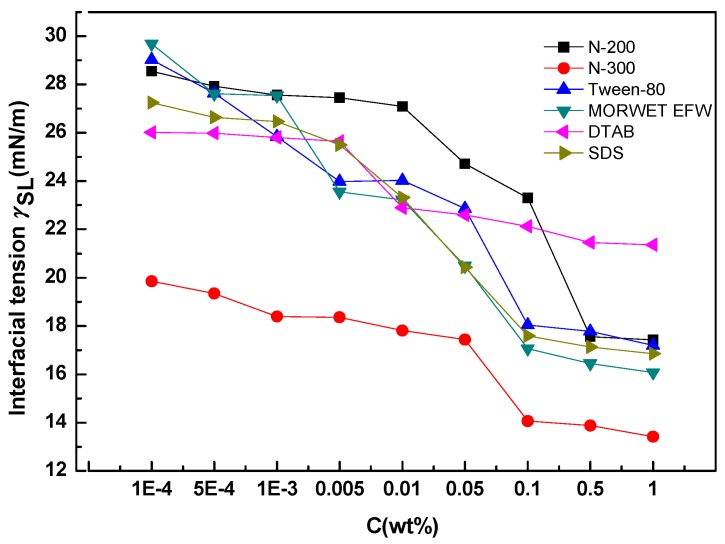
Relationship between interfacial tension (*γ*_SL_) and surfactant concentrations.

As shown in Figure 7, the interfacial tension of all surfactants on tea leaf surfaces changed with variations in the concentration of surfactant solutions. The interfacial tension decreased with increasing concentrations of surfactant solutions until it reached a balance phase when the concentration was 0.1%. By comparing the differences of these testing surfactants, it was clear that they varied from each other. As for N-300 and DTAB, the interfacial tension dropped slowly throughout the process. Concerning the other four surfactants, the interfacial tension dropped gradually at first, but as concentrations reached 0.001%, the interfacial tension decreased sharply. Finally, it reached a plateau. Still, we found that it was more difficult to evaluate the absorbing behavior of a surfactant on plant leaf surfaces compared to abiotic surfaces such as polytetrafluoroethylene (PTFE) [27].

### 3.5. Structural Dependence of Surfactant Solutions on the Wettability of Tea Leaf Surfaces

**Figure 8:**
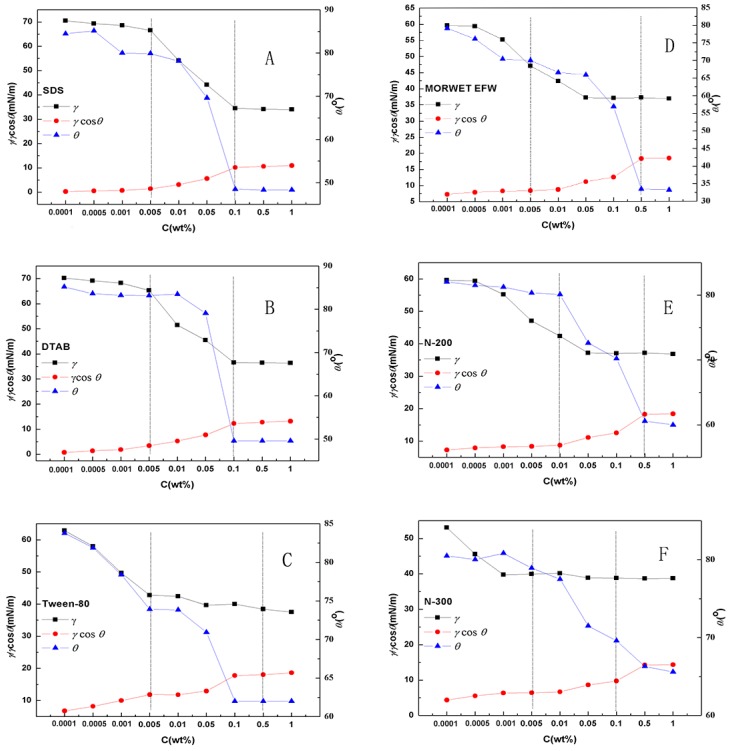
Dependence of the adsorption data of SDS (**A**), DTAB (**B**), Tween-80 (**C**), Morwet EFW (**D**), N-200 (**E**), and N-300 (**F**) on tea leaf surfaces.

Differently from the smooth solid surface of PMMA, which is in accordance with Young’s state, the wetting behavior of surfactants on tea leaf surfaces is complicated due to the rough surfaces of the leaves. For cases of rough surfaces, the Wenzel equation and Cassie–Baxter model were introduced to explain the wetting properties. In order to evaluate the wetting state and wetting behavior mechanism of aqueous surfactant solutions on tea leaf surfaces, the dependence of the contact angle and *γ_LV_*cos*θ* on the concentration were replotted, as shown in Figure 8. The variations with increasing mass concentrations of aqueous surfactant solutions on tea leaf surfaces could be divided into three stages for all surfactants.

As shown in Figure 8, SDS (A), DTAB (B), N-200 (E), and N-300 (F) showed similar behaviors on tea leaves. In the first stage (from 0.0001% to 0.001%), the contact angle was maintained at a constant value because the concentration was too low to form adsorption films at both the liquid–air and liquid–solid interfaces. During this stage, the surface tension and the adhesion tension were almost unchanged because the adsorption films were not yet formed.

In stage 2 (from 0.005% to 0.1%), the surface tension and adhesion tension decreased sharply. This may have been caused by the unsaturated adsorption films that were formed by the surfactant [10]. At the same time, the contact angle decreased sharply. Furthermore, the absorption volume of the surfactant molecules on the tea leaf surfaces increased significantly.

In stage 3 (from 0.1% to 1%), both the surfactant molecules at the liquid–air and solid–liquid interfaces reached the absorption balance. In this case, the surface tension, adhesion tension, and contact angle did not change with an increase in the concentration.

As shown in Figure 8C,D, Tween-80 (C) and Morwet EFW (D)exhibited similar wettability, but some differences still existed.

In stage 1 (from 0.0001% to 0.005%), the contact angle decreased gradually with an increase in the concentration. In addition, the surface tension decreased due to the formation of adsorption films.

In stage 2 (from 0.005% to 0.5%), the contact angle of surfactant C decreased sharply, but during this stage, the surface tension was maintained almost at a constant value, while the adhesion tension increased gradually. Compared to surfactant C, the contact angle of surfactant D remained constant. However, the surface tension decreased gradually, while the adhesion tension increased gradually.

In stage 3 (from 0.5% to 1%), when the concentration reached its CMC, because there was no adsorption on tea leaf surfaces, the contact angle, surface tension, and adhesion tension were all kept at constant values.

### 3.6. Analysis of Adhesion Work

The adhesion work (*W*_a_) of surfactants could be calculated using Equation (6):

(6) $$W_{a}=\gamma_{SV}+\gamma_{LV}-\gamma_{SL}$$

Substituting this equation into Young’s equation, the adhesion work could be obtained:

*W*_a_ = *γ_LV_*(1 + cos*θ*) (7)

**Figure 9:**
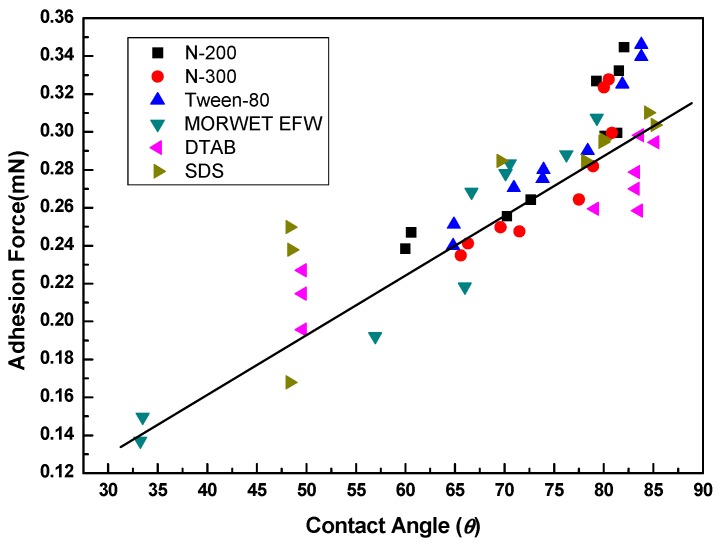
Relationship between the adhesion force (mN) and contact angle (*θ*) of aqueous surfactant solutions on tea leaf surfaces.

By inserting the contact angle and surface tension of the aqueous surfactant solution into Equation (7), the *W*_a_ of the solution on tea leaf surfaces could be obtained. The relationship between *W*_a_ and the concentration of the surfactants is presented in Figure 9. This figure suggests that the values of *W*_a_ of DTAB and SDS remained constant under a mass concentration of 0.005%. With an increase in the surfactant concentration, *W*_a_ decreased sharply until the concentration reached up to 0.1%, after which *W*_a_ was maintained at a constant level. As for N-200, Tween-80, and Morwet EFW, all of their values of *W*_a_ decreased with an increase in the mass concentration. When their mass concentrations reached 0.05%, their *W*_a_ decreased to the lowest point, which was the minimum value of *W*_a_. Subsequently, their *W*_a_ increased with the concentration until their concentrations reached 0.5%, after which *W*_a_ was kept at a constant value. For N-300, *W*_a_ decreased to the minimum value at a concentration of 0.001%, then increased slightly and finally arrived at a constant value. The evaluation of *W*_a_ was an easy and valuable method that can be used to detect the degree of adhesion for a solid surface. Due to the strong correlation between adhesion and the mechanism of adhesion, the amount of agrochemical solution that should be deposited on the plant leaf surface can be presented by *W*_a_ [28].Generally, a high wettability is paired with the highest *W*_a_: As *W*_a_ increases, the level of hydrophobicity weakens, so more work is required to separate the liquid and solid phases [29].

### 3.7. Relationship Between Adhesion Force and Contact Angle (CA)

The contact angle is a traditional parameter used to characterize the hydrophobicity or hydrophilicity of a solid surface. As shown in Figure 9, the adhesion force increased with an increase in the contact angle, which differs from many plant surfaces. In many cases, a high contact angle is always coupled with a low adhesion force. As a result, water droplets can roll down from a solid surface, a typical phenomenon defined as the “lotus effect”. Compared to the lotus effect, which is widely observed in nature, a similar phenomenon occurs, wherein a high contact angle can coexist with strong adhesion between water and a solid surface: The so-called “rose petal effect” [30]. Even for rose petals, a superhydrophobic surface with high adhesion can still coexist with low adhesion. The adhesion of rose petal surfaces has a strong correlation with the nanostructure and microstructure of leaf surfaces [31,32]. In contrast, peanut leaves exhibit both superhydrophobicity and a high adhesive force, which is different from lotus leaves with low adhesive superhydrophobicity because water droplets do not slide down from peanut leaf surfaces [32]. Interestingly, tea leaf surfaces are not smooth to the naked eye, but the undulating surfaces do not repel water drops. Moreover, the lack of micro and nano structure on tea leaf surfaces may lead to the coexistence of a high contact angle and a high adhesion force.

### 3.8. Maximum Retention Analysis

**Figure 10:**
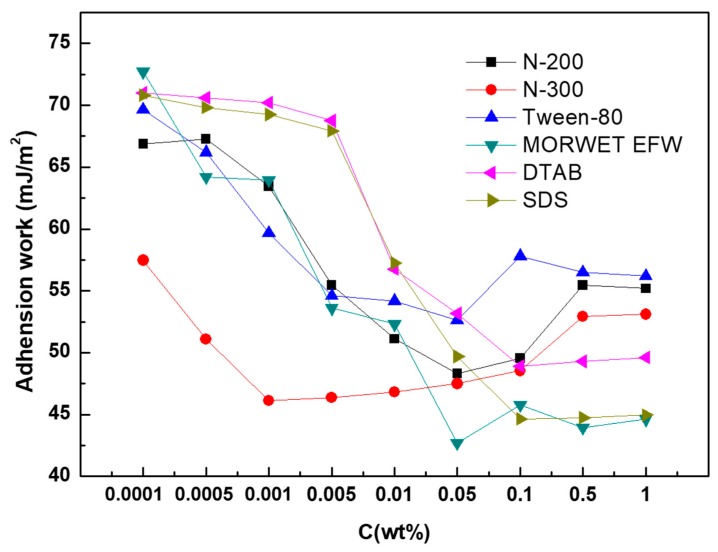
Dependence of the adhesion work of aqueous surfactant solutions on tea leaf surfaces.

**Figure 11:**
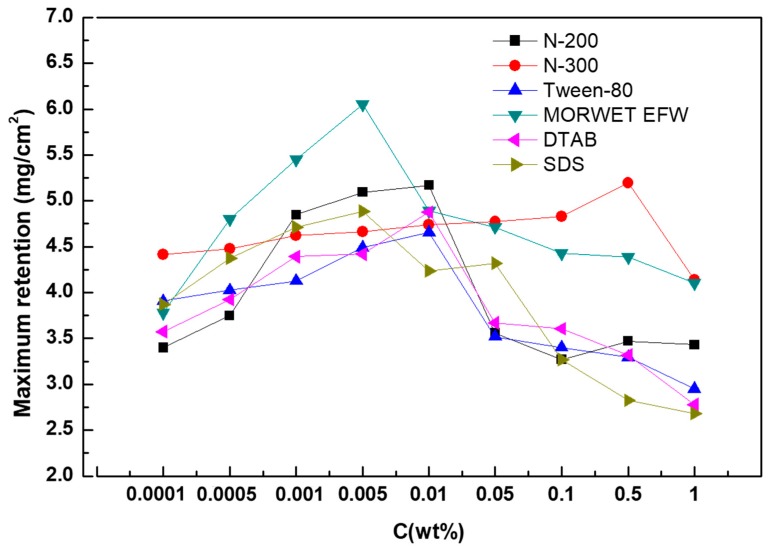
Dependence of the maximum retention of aqueous surfactant solutions on tea leaf surfaces.

It is supposed that tea leaves are difficult to wet due to their glossy surfaces. However, in this study, our results revealed that the maximum retention of surfactant solutions could be observed before reaching the CMC. The maximum retention of N-200 and DTAB was at 0.01%, while that of Morwet EFW and SDS was at 0.005%. It suggested that using Morwet EFW can enable the highest dose of solution on leaves compared to other surfactant solutions. In addition, a high concentration did not mean more retention on tea leaves. As seen in Figure 10 and Figure 11, with a decrease in adhesion work, the retention of surfactant solutions on tea leaf surfaces was increased, but the liquid was still running off of leaf surfaces after the maximum retention was reached.

## 4. Conclusions

In the present work, in order to understand the wettability of six kinds of commercial surfactants on tea leaf surfaces, the contact angle, adhesion force, adhesion work, surface free energy, and adhesion tension were measured. The results showed that with an increase in the concentration, the contact angle on tea leaf surfaces decreased, and then it reached a constant in the end. In addition, both *γ_LV_*cos*θ* and the interfacial tension exhibited similar variation tendencies with changes in the contact angle. To understand the wetting mechanisms of surfactants on tea leaf surfaces, the wetting process can be explained by the adsorption of surfactants on tea leaf surfaces. When the concentration was lower than 0.005%, the contact angle almost remained constant, suggesting that adsorption at the solid–liquid and liquid–air interfaces was too low. With an increase in the concentration of surfactants, better surface activity was achieved, so the contact angle decreased sharply.

It is not easy to evaluate the wettability of biotic surfaces, so adhesion work was introduced to explain the adhesion degree between the surfactant solutions and the tea leaf surfaces. With an increase in the concentration, the adhesion work decreased sharply, reaching a minimum at the CMC, but then increased until it reached a constant. Moreover, high adhesion force did not indicate better wettability, as it does in rose petals and peanut leaves. For tea leaf surfaces, the contact angle increased with an increase in the adhesion force. However, this is different from peanut leaves, where water cannot slide down from leaf surfaces. This is mainly because of the lack of microstructures and nanostructures, such as deep pitches on the leaf surfaces. Furthermore, the maximum retention of surfactant solutions on tea leaves increased with an increase in the concentration, but the surfactant solutions dropped off when the tea leaves reached the point of spraying run-off.

Generally, surfactants can be used to improve the properties of pesticides to meet the demands of reducing the use of pesticides for plant protection. The efficiency of pesticides can be affected by many factors due to the growth conditions of agricultural plants, but wettability is one of the key influencing factors. Unlike ideal solid surfaces, plant leaf surfaces are more complicated, making it difficult to characterize their wettability, especially in tea leaves. Typically, the raw leaves of tea are used to prepare a drink after simple processing, while the edible parts of most agricultural plants are fruits. In addition, the appropriate amount of pesticide solution that sticks to the tea leaves should be taken into consideration. Therefore, throughly understanding of the wetting behavior of surfactants on tea leaf surfaces is very crucial. This work may provide the potential to improve pesticide efficiency by adjusting the wettability of exclusive pesticide formulations so as to minimize the adverse effects of pesticide son the environment and public health.

## Figures and Tables

**Table 1:** Information for six surfactants.

Chemicals	Description	Producer	Activity (%)
N-200	Acrylate homologues	Dauni Research Center of Advanced Science and Technology Co., Ltd.	37.0
N-300	Aliphatic alcohol polyoxyethylene ethers	Dauni Research Center of Advanced Science and Technology Co., Ltd.	95.0
Morwet EFW	Blend of alkyl naphthalene sulfonate and anionic wetting agent	Azkonobel	95.0
Tween-80	Polyethylene glycol sorbitan monooleate	Solarbio Life Science	70.0
DTAB	Dodecyl trimethyl ammonium bromide	Sigma-Aldrich Co., Ltd.	98.0
SDS	Sodium dodecyl sulfate	Sigma-Aldrich Co., Ltd.	99.0

**Table 2:** Surface free energy, dispersion component, and polar component of probe liquids on tea leaf surfaces.

Probe Liquids	Surface Free Energy (MJ/m^2^)	Dispersion Component (MJ/m^2^)	Polar Component (MJ/m^2^)
Deionized water (Chen et al. [18])	72.8	29.1	43.7
Formamide (Chen et al. [18])	58.2	35.1	23.1
Ethyleneglycol (Janczuk et al. [19])	48.2	29.29	18.91
*N*,*N*-Dimethylformamide [20] (Fowkes)	37.3	32.42	4.88

**Table 3:** Surface free energy and other components of tea leaf surfaces.

	Surface Free Energy (MJ/m^2^)	Dispersion Component (MJ/m^2^)	Polar Component (MJ/m^2^)	Proportion of Dispersion Component (%)	Proportion of Polar Component (%)
Tea leaves	30.4	28.67	1.73	94.3	5.7

**Table 4:** Slope and coefficient values obtained by the relationship between the surface tension and adhesion tension.

Surfactant	Stage 1		Stage 2	
	Slope	Coefficient	Slope	Coefficient
N-200	/	/	−0.042	0.8624
N-300	/	/	−0.15	0.9972
Tween-80	−0.24	0.9973	−0.25	0.9929
Morwet EFW	/	/	−0.23	0.9888
DTAB	/	/	−0.54	0.9975
SDS	/	/	−0.17	0.9667

## Supplementary Materials

The following are available online at https://www.mdpi.com/1420-3049/24/11/2094/s1, Figure S1: Dynamic surface tensions for SDS solutions.
