# Supplementary figures and images for: Wetting Behavior and Maximum Retention of Aqueous Surfactant Solutions on Tea Leaves
Source: Molecules. 2019 Jun 1;24(11):2094. doi: 10.3390/molecules24112094 (PMC6600537; doi:10.3390/molecules24112094)

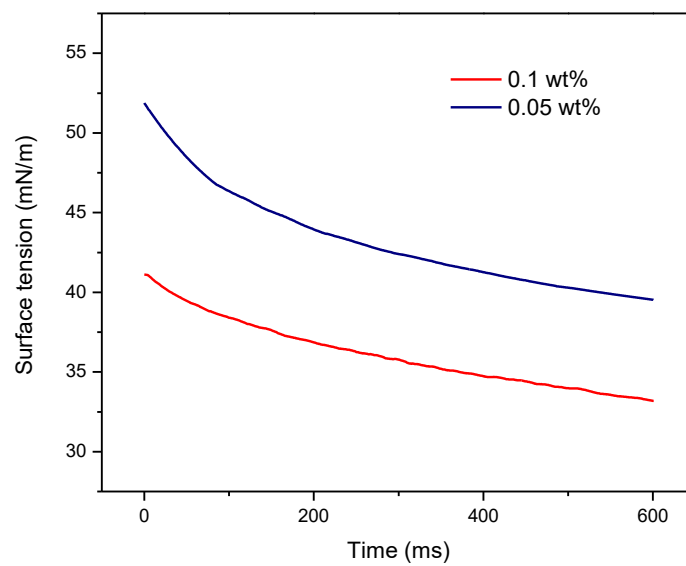

**Figure S1.** Dynamic surface tensions for SDS solutions.

Supplement: Supplementary file 1 [file molecules-24-02094-s001.pdf]
